# Regorafenib enhances anti-PD1 immunotherapy efficacy in murine colorectal cancers and their combination prevents tumor regrowth

**DOI:** 10.1186/s13046-021-02043-0

**Published:** 2021-09-13

**Authors:** Dennis Doleschel, Sabine Hoff, Susanne Koletnik, Anne Rix, Dieter Zopf, Fabian Kiessling, Wiltrud Lederle

**Affiliations:** 1 Institute for Experimental Molecular Imaging, Medical Faculty, RWTH Aachen University, Aachen, Germany; 2 Research and Development, Preclinical Research Oncology, Bayer AG, Berlin, Germany; 3 Fraunhofer Institute for Digital Medicine MEVIS, Bremen, Germany

**Keywords:** Colorectal cancer, Metastasis, Anti-PD1 antibody, Regorafenib, Preclinical, Macrophage polarization

## Abstract

**Background:**

Patients with advanced colorectal cancer (CRC) have a poor prognosis. Combinations of immunotherapies and anti-angiogenic agents are currently being evaluated in clinical trials. In this study, the multikinase inhibitor regorafenib (REG) was combined with an anti-programmed cell death protein 1 (aPD1) antibody in syngeneic murine microsatellite-stable (MSS) CT26 and hypermutated MC38 colon cancer models to gain mechanistic insights into potential drug synergism.

**Methods:**

Growth and progression of orthotopic CT26 and subcutaneous MC38 colon cancers were studied under treatment with varying doses of REG and aPD1 alone or in combination. Sustained effects were studied after treatment discontinuation. Changes in the tumor microenvironment were assessed by dynamic contrast-enhanced MRI, and histological and molecular analyses.

**Results:**

In both models, REG and aPD1 combination therapy significantly improved anti-tumor activity compared with single agents. However, in the CT26 model, the additive benefit of aPD1 only became apparent after treatment cessation. The combination treatment efficiently prevented tumor regrowth and completely suppressed liver metastasis, whereas the anti-tumorigenic effects of REG alone were abrogated soon after drug discontinuation. During treatment, REG significantly reduced the infiltration of immunosuppressive macrophages and regulatory T (Treg) cells into the tumor microenvironment. aPD1 significantly enhanced intratumoral IFNγ levels. The drugs synergized to induce sustained M1 polarization and durable reduction of Treg cells, which can explain the sustained tumor suppression.

**Conclusions:**

This study highlights the synergistic immunomodulatory effects of REG and aPD1 combination therapy in mediating a sustained inhibition of colon cancer regrowth, strongly warranting clinical evaluation in CRC, including MSS tumors.

**Supplementary Information:**

The online version contains supplementary material available at 10.1186/s13046-021-02043-0.

## Background

Colorectal cancer (CRC) is one of the main causes of cancer-related death worldwide [1, 2]. Despite significant improvement in early cancer detection, most cases of CRC are still diagnosed at the advanced stages of the disease. For advanced metastatic CRC, the 5-year survival rate after diagnosis is approximately 10% [3], thus emphasizing the need for novel therapeutic options to improve patient survival. For many years, the only approved first- and second-line treatment options for CRC were surgical interventions followed by chemotherapy regimens and combinations with antibodies against epidermal growth factor receptor (EGFR) or vascular endothelial growth factor (VEGF) if indicated [4]. Since 2012, the therapeutic landscape has been moderately improved by the approval of the multikinase inhibitor regorafenib (REG) and TAS-102 (trifluridine/tipiracil; approved in 2015). REG and TAS-102 each increased overall survival when used as single-agent treatments for patients with CRC for whom previous chemotherapy regimens had failed [5].

REG is an oral, Small-molecule inhibitor that potently blocks multiple protein kinases, including VEGF receptor (VEGFR) 1, 2, and 3, TIE2, KIT, RET, RAF1, BRAF, BRAFV600E, platelet-derived growth factor receptors, fibroblast growth factor receptor, and colony stimulating factor 1 receptor (CSF1R) [6, 7]. REG has potent anti-angiogenic and immunomodulatory properties, as previously demonstrated by the strong reduction of macrophages in orthotopic CT26 colon cancer xenografts [6, 8, 9]. REG is approved as a single agent therapy for advanced and pre-treated CRC, gastrointestinal stromal tumors, and hepatocellular carcinoma (HCC) [10–14].

In addition, immune checkpoint inhibition is a promising, novel therapeutic strategy aiming to stimulate anti-tumor immunity in patients. Pembrolizumab and nivolumab—antibodies targeting programmed cell death protein 1 (PD1)—are approved as monotherapies or, in the case of nivolumab, in combination with the anti-cytotoxic T lymphocyte-associated protein 4 antibody ipilimumab for the treatment of CRC with high microsatellite instability (MSI-H)/mismatch repair deficiency (dMMR) [15]. PD1 is expressed on T cells and binds to two receptors, PD-L1 and PD-L2, which are expressed by tumor and immune cells. Since binding of PD1 to PD-L1 suppresses effector T cell activation and induces T cell exhaustion, blockade of this pathway using anti-PD1/PD-L1 antibodies can reactivate T cells and restore their effector functions, thus enhancing the anti-tumor immunity [16]. Unfortunately, most CRC patients respond poorly to single-agent immunotherapy, if at all, due to the high prevalence of mismatch repair-proficient (pMMR) and microsatellite-stable (MSS) tumors [17]. Thus, combination therapies of immune checkpoint inhibitors (ICIs) with other anti-cancer drugs, including anti-angiogenic agents, for patients with pMMR/MSS CRC are currently being explored in clinical trials [18]. Early clinical data reported from small cohorts of pMMR/MSS CRC patients treated with various ICI and antiangiogenic drug combinations revealed heterogeneous outcomes, and are not yet conclusive with respect to their efficacy [19–22]. The most encouraging anti-tumor activity was observed in a phase Ib trial, in which the combination of REG and nivolumab showed an objective response rate of 33% in patients with MSS/pMMR CRC previously treated with at least two lines of therapy [23]. To gain further insight into the anti-tumor and immunomodulatory effects as well as the underlying mechanisms of action, we investigated REG in combination with anti-PD1 (aPD1) in two murine CRC models—the subcutaneous MC38 hypermutated/MSI tumor model and the orthotopic CT26 non-hypermutated/MSS tumor model [24, 25].

## Materials and methods

### Therapeutic agents

REG, provided by Bayer AG, was dissolved in propylene glycol/PEG400/Pluronic F68 (42.5/42.5/15 + 20% aqua) for in vivo applications. Anti-murine PD1 (aPD1, catalog #BE0146, clone RMP1-14) and isotype rat immunoglobulin gamma 2A (ISO, catalog #BE0089) antibodies were purchased from BioXCell and dissolved in PBS. Therapies were well tolerated and decreases in animal body weight did not exceed 10% of body weight at the start of treatment (data not shown).

### Cell lines and cell culture

The murine MC38 colon adenocarcinoma cell line was originally derived from C57BL/6 mice treated with the carcinogen 1,2-dimethylhydrazine [26]. Cells were cultivated in RPMI 1640 with 10% fetal calf serum, 10 mM HEPES buffer, and non-essential amino acids.

The murine CT26 grade IV colon carcinoma cell line (LGC Standards GmbH) was originally derived from BALB/c mice treated with the carcinogen N-nitroso-N-methylurethane [27]. Cells were cultivated in Dulbecco’s Modified Eagle’s Medium (Gibco, Invitrogen GmbH) containing 10% FBS (Gibco) and 1% penicillin/streptomycin (Gibco) at 37 °C and 5% CO_2_.

Cell lines were maintained in culture for no longer than 6 months, and mycoplasma contamination was excluded by Hoechst staining, polymerase chain reaction or enzymatic test (MycoAlert, Lonza) prior to in vivo application.

### In vivo experiments

Mouse experiments were approved by the regulatory agencies of the German Federal States of Berlin (Federal Office for Health and Social Affairs) and North Rhine-Westphalia (Authority for Nature, Environment and Consumer Protection).

### MC38 syngeneic subcutaneous CRC model

1 × 10^5^ MC38 cells in medium were injected subcutaneously with 50% Matrigel into the flank of 6–8-week-old female C57BL/6 N mice (Charles River) and tumors were grown to approximately 60 mm^3^. Mice were randomized to four groups (*n* = 10 each); each mouse was treated with REG 3 mg/kg once daily by oral gavage or aPD1 10 mg/kg every third day by intraperitoneal (i.p.) injection for a total of five doses, or with the combination of both for a duration of 15 days. The combination of vehicle (VEH) + ISO served as the control group. Minor and major axes (d and D) of the tumor were measured by caliper three times per week, and volumes were calculated using the formula (D × d^2^)/2. After 15 days of treatment, mice were sacrificed; tumors were recovered and snap frozen.

### CT26 syngeneic orthotopic CRC model

Orthotopic tumors were generated as previously described [9]. In brief, 1 × 10^6^ CT26 cells in culture medium were injected subcutaneously into the right flank of 6–8-week-old female BALB/cAnNRj donor mice (Janvier). These mice were euthanized 10 days post-injection when tumors had reached a size of approximately 500 mm^3^. Tumors were excised, cut into fragments of 1–2 mm, and collected in ice-cold PBS; necrotic areas were removed. For orthotopic implantation, mice received carprofen 5 mg/kg of body weight 2 h before surgery and were anesthetized with isoflurane (2%) immediately before surgery. After shaving and disinfection of the abdomen (Antiseptica), mice underwent laparotomy (0.5 cm in size), and one tumor fragment was implanted into the cecum as described previously [9]. After surgery, carprofen was administered every 12 h, and mice were allowed to recover for 4 days before initiation of therapy and measurements.

### Efficacy study in orthotopic CT26 tumors

For longitudinal analyses of tumor growth and progression in response to continuous therapy for 10 days, mice were randomized into six treatment groups with a minimum of six animals per group on day 4 post-implantation. Mice were treated daily by oral gavage with REG (30 mg/kg body weight; group 1) or VEH (group 2), as described previously [9]. Mice received i.p. injection of aPD1 (20 mg/kg) or received ISO (20 mg/kg) every third day in groups 3 and 4, respectively. Mice in group 5 received REG (30 mg/kg body weight, orally daily) and aPD1 (20 mg/kg, i.p., every third day). Animals in group 6 were treated with VEH (orally, daily) and ISO (20 mg/kg, every third day, i.p.).

Therapy was continued for 10 days until day 14 post-implantation. Animal weight was measured daily. Tumor volumes and tumor vascularization were measured longitudinally by MRI on days 4, 7, 11, and 14 after implantation. On day 14, animals were euthanized after MRI measurements. Tumors were dissected and cryopreserved for histological analyses. In addition, the liver of each mouse was resected and macroscopically screened for metastases.

For histological analyses of mice at intermediate timepoints during the longitudinal study, additional mice with orthotopic tumors were sacrificed on day 4 after implantation (before therapy initiation), or received treatment as described earlier and were sacrificed on day 7 and 11 (5 mice per group).

### Post-therapeutic progression study in orthotopic CT26 tumors

For the analysis of tumor progression after therapy discontinuation, seven mice per group were treated with either REG (30 mg/kg, orally daily) and ISO (20 mg/kg, i.p., every third day), or with REG (30 mg/kg, orally daily) and aPD1 (20 mg/kg, i.p., every third day). Therapy was administered for 10 days until day 14 post-implantation, and tumor volumes and vascularization were longitudinally measured by MRI, including dynamic contrast-enhanced MRI (DCE-MRI) on days 4, 7, 11, and 14 post-implantation. Subsequently, therapy was stopped, and tumor growth and vascularization were further monitored by MRI on days 18, 21, and 25 post-implantation. Animal weight was measured daily during the entire observation period. On day 25 post-implantation, animals were sacrificed after MRI measurements. As described earlier, tumors were resected and cryopreserved for histological analyses. In addition, the liver of each mouse was dissected and macroscopically screened for metastases.

### MRI measurements

MRI analyses were performed using a preclinical small animal MR scanner (Bruker ICON 1 T). For scanning, mice were anesthetized (isoflurane 2% v/v) and kept at a constant temperature (37 °C).

Tumors were localized and the sizes determined using T1- and T2-weighted Rapid Acquisition with Relaxation Enhancement (RARE) spin echo sequences (details on sequences see Supplementary methods). Tumor volumes were calculated using the Imalytics Preclinical software (Gremse-IT GmbH) and normalized to the tumor volume before therapy initiation to obtain relative tumor volume changes.

Tumor vascularization was analyzed longitudinally using DCE-MRI measurements. For DCE-MRI, a T1-weighted saturation recovery Fast Low Angle Shot (FLASH) sequence was applied (details on sequence see Supplementary methods). In total, 80 sequential images were acquired per slice with a temporal resolution of 7.9 s, resulting in a total scan time of 10.53 min. After the acquisition of baseline images over approximately 2 min, 80 µl (100 µmol/kg body weight) of the paramagnetic contrast agent Gadomer 17 (invivoContrast GmbH) was injected into the tail vein. Kinetic modeling was performed as described previously [9]. In brief, the average signal per region was computed and the resulting signal–time curves were analyzed using the pharmacokinetic two-compartment model of Brix and colleagues [28, 29] and DynaLab software (Fraunhofer MEVIS). The parameter amplitude A relates to the relative distribution volume of the contrast agent in the tumor which usually also relates to the relative blood volume [30]. Phantom experiments were performed in advance to confirm the linearity between the applied contrast agent concentrations and signal intensities. For longitudinal analysis of tumor vascularization, the amplitude values were normalized to the initial values before treatment start to obtain relative amplitude values.

### Indirect immunofluorescence

Tumors were resected, frozen in Tissue-Tek O.C.T. (Sakura Finetek), and sliced into 7–10 μm sections. Fixation of frozen sections and the staining procedure were performed as described previously [9]. Specifications about primary and secondary antibodies are provided in Supplementary methods. The stained sections were visualized using an epifluorescence microscope (Axio Imager M2, Zeiss) equipped with a high-resolution camera (AxioCamMRmRev.3, Zeiss) covering representative areas for each tumor. AxioVision Rel 4.8 (Zeiss) and ImageJ 1.50i (National Institutes of Health) software were used for image quantification.

### Ex vivo analyses of tumor samples

For assessing IFNγ protein levels, snap-frozen tumor samples were lysed in MSD Tris Lysis Buffer (MSD) using a TissueLyser II (Qiagen) and stainless-steel beads (5 mm in diameter; Qiagen). The lysates were centrifuged at 25,000 × g at 4 °C for 20 min, protein concentrations were determined using the Bradford assay, and 400 µg supernatants were analyzed for IFNγ concentration by enzyme-linked immunosorbent assay using the V-PLEX Proinflammatory Panel 1 Mouse Kit (MSD).

For the IFNγ mRNA expression analysis, snap-frozen tumor samples (20–30 mg, *n* = 5 tumor samples/group) were lysed using the Precellys Ceramic Kit (#91-PCS-CK28; Bertin Corporation), followed by total RNA extraction using the RNeasy Plus Mini Kit (Qiagen) and cDNA synthesis using the iScript cDNA Synthesis Kit (Bio-Rad) following the manufacturer’s instructions. Gene expression analysis was performed by quantitative real-time PCR (qRT-PCR) in a 384-well plate (MicroAmp® Optical 384-Well Reaction Plate, Applied Biosystems) using a 7900HT Fast RT-PCR System (Applied Biosystems). The TaqMan PCR reaction was prepared in 10 μL containing 15 ng cDNA and the probes for *IFNγ* (probe ID: Mm01168134_m1) and for *GAPDH* (probe ID: Mm99999915_ g1) using 2 × TaqMan Universal Master Mix (Thermo Fisher Scientific) following the manufacturer’s instructions. Relative mRNA expression was calculated using the ΔΔC_T_ method, using endogenous *GAPDH* mRNA expression as reference. Expression is depicted as fold induction over relative expression in the VEH- and ISO-treated control groups.

### Statistical analysis

Statistical analyses were performed using GraphPad Prism software (version 7 or newer). Statistical significance was estimated by one-way analysis of variance with Tukey’s post-test for multiple comparisons, using log_10_-transformed values in the case of the MC38 experiment. A Šidák post-test was used for CT26 experiments. Several control groups were utilized in these experiments along with separate control versus treatment comparisons. In the post-therapeutic progression study, statistical significance was estimated with a one-way t-test using log_10_ transformed values. *P* values < 0.05 were considered statistically significant. Data are expressed as mean ± SD.

## Results

### The combination of REG and aPD1 leads to additive tumor growth inhibition in subcutaneous MC38 MSI tumors

**Fig. 1:**
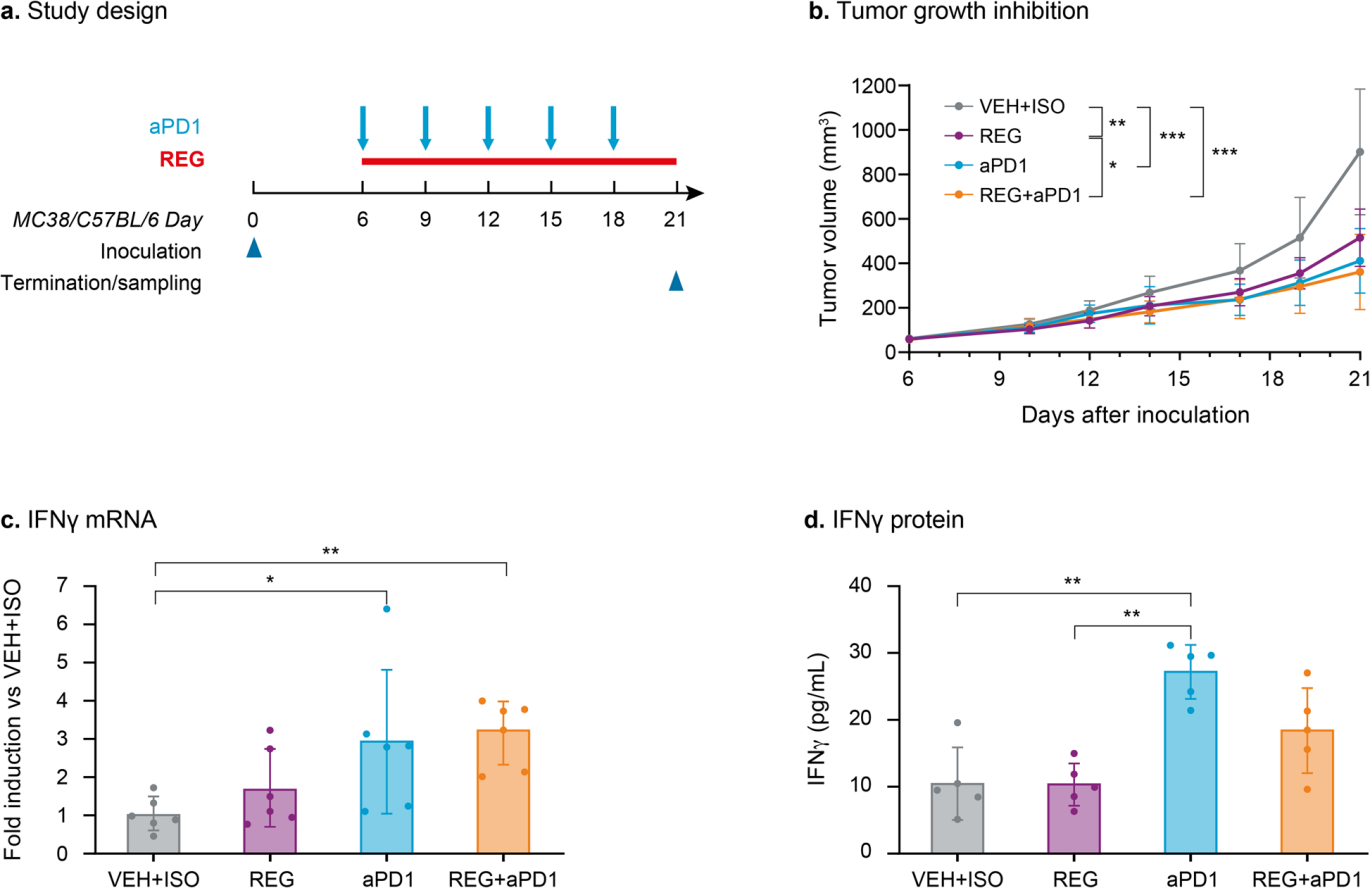
The combination of REG and aPD1 results in additive suppression of subcutaneous MC38 tumor growth. **a** Study design. **b** Tumor growth curves with mean tumor volumes ± SD (*n* = 10). **c** Intratumoral levels of IFNγ mRNA at study end determined by qRT-PCR. **d** Intratumoral levels of IFNγ protein from total tumor lysates at study end determined by enzyme-linked immunosorbent assay. Mean ± SD (*n* = 5) and individual values (dots) are shown. *p < 0.05; **p < 0.01; ***p < 0.001

We first investigated the anti-tumor activity of REG in combination with aPD1 (REG + aPD1) in a syngeneic subcutaneous MC38 MSI colon cancer model (Fig. 1a). To detect a potential treatment benefit of this combination, REG was dosed at 3 mg/kg daily, which previously induced an intermediate tumor growth inhibition of approximately 50% versus control (data not shown). All treatments significantly inhibited tumor growth in comparison with the VEH + ISO group. REG 3 mg/kg + aPD1 significantly reduced tumor growth versus REG alone but did not reduce growth versus aPD1 alone (Fig. 1b).

IFNγ levels were measured in the tumors at the end of the study to assess the possible mechanism of action. REG did not affect the levels of IFNγ mRNA or protein in comparison with the control (Fig. 1c and d). However, aPD1 markedly increased IFNγ mRNA and protein levels, which mechanistically confirms that aPD1 is effective in reactivating cytotoxic T cells [31]. The combination of REG + aPD1 had no additional effect on IFNγ levels in the tumors compared with aPD1 alone.

### REG and REG + aPD1 significantly inhibit tumor growth and liver metastasis in orthotopic CT26 MSS colon tumors

**Fig. 2:**
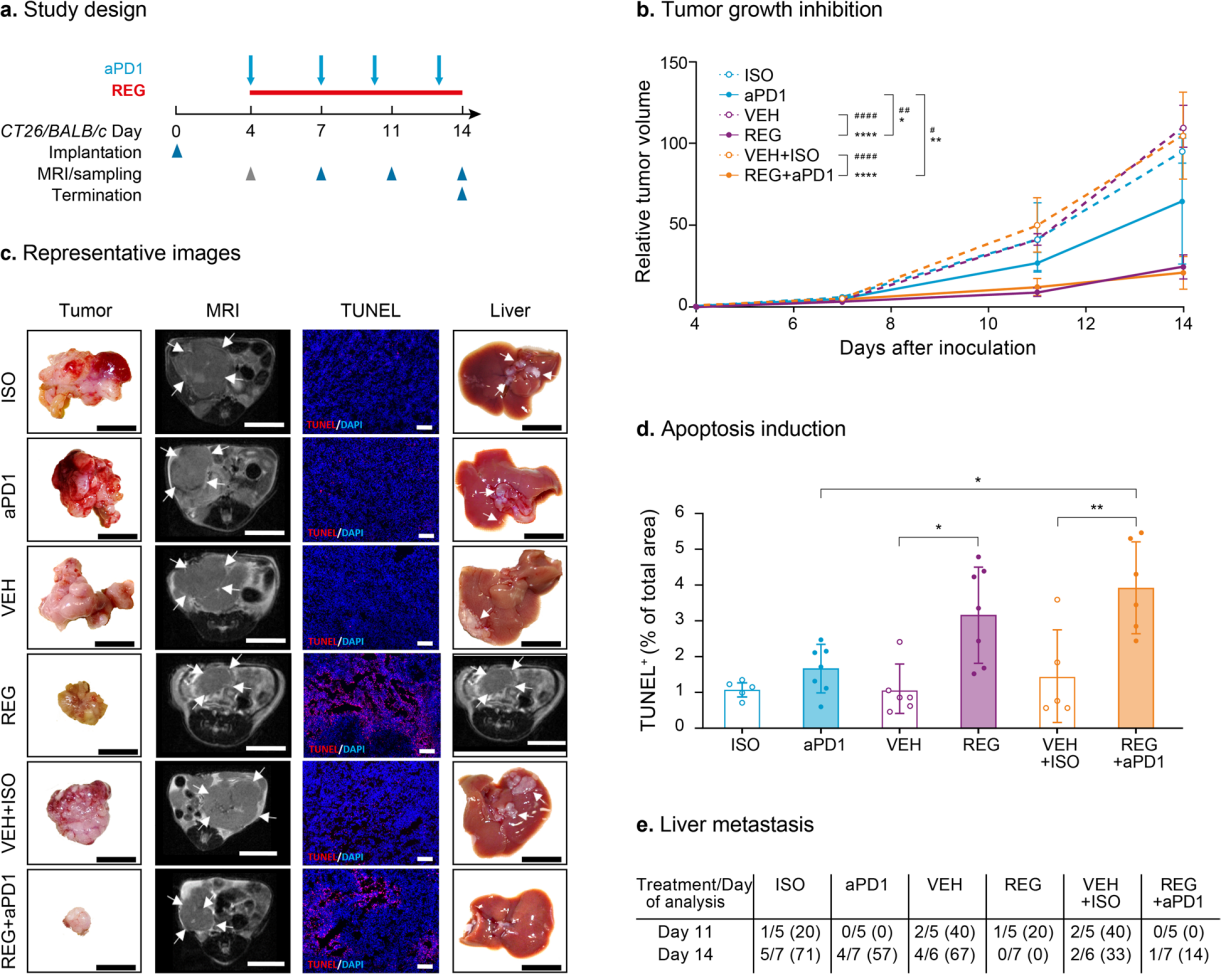
REG and REG + aPD1 significantly inhibit tumor growth and liver metastasis in orthotopic CT26 colon tumors. **a** Study design. Gray arrowhead indicates imaging/sampling before first drug administration for baseline determination. **b** Tumor growth inhibition. Mean RTV given as a percentage of the baseline volumes ± SD (*n* = 6–7). **c** Representative images. First panel: dissected tumors; second panel: T2-weighted MR images (white arrows demarcate tumors); third panel: TUNEL staining (red) with DAPI stained nuclei (blue); fourth panel: livers with metastases depicted by white arrows. Scale bar: 10 mm (MRI and ex vivo) and 100 µm (TUNEL). **d** Quantification of TUNEL stainings (tumor apoptosis) from frozen tumor sections at study end with mean values ± SD (*n* = 5–7) and individual values (dots). Statistical significance (^#^day 11; *day 14): *^/#^*p* < 0.05; **^/##^*p* < 0.01; ***^/###^*p* < 0.001. **e** Number of mice with liver metastases compared with total number of mice and percentages (number in %)

We also studied REG + aPD1 in the syngeneic CT26 MSS colon cancer model [24, 25, 32] (Fig. 2a). Tumors were orthotopically implanted into the cecum wall as described previously [9], and mice were treated with either REG or aPD1 alone or received REG + aPD1 until day 14 post-implantation. REG was dosed at 30 mg/kg, a previously used dose demonstrating anti-tumor efficacy in orthotopic xenograft tumors [9]. Controls included mice treated with VEH, ISO, and VEH + ISO. MRI was used to monitor tumor growth over time. REG and REG + aPD1 strongly inhibited tumor growth to a similar extent, while only modest tumor growth inhibition was observed with aPD1 alone (Fig. 2b, c panels “tumor” and “MRI”; see Supplementary Figure S1 for tumor growth curves of individual mice).

In line with the strong inhibitory effect on tumor growth, a significantly higher proportion of cells undergoing apoptosis was observed in tumors treated with REG and REG + aPD1 compared with control tumors at day 14 post-implantation. aPD1 alone induced only a marginal degree of tumor apoptosis (Fig. 2d, c panel “TUNEL”).

Macroscopic inspection of dissected organs identified liver metastases starting at day 11 after tumor implantation. Metastases were most frequently detected in control animals. By day 14 post-implantation, aPD1 alone had moderately reduced the liver metastases, whereas REG alone and REG + aPD1 almost completely prevented their occurrence (Fig. 2e, c panel “liver”).

### DCE-MRI and histological analyses demonstrate the strong inhibitory effects of REG on tumor angiogenesis in the orthotopic CT26 MSS colon cancer model

**Fig. 3:**
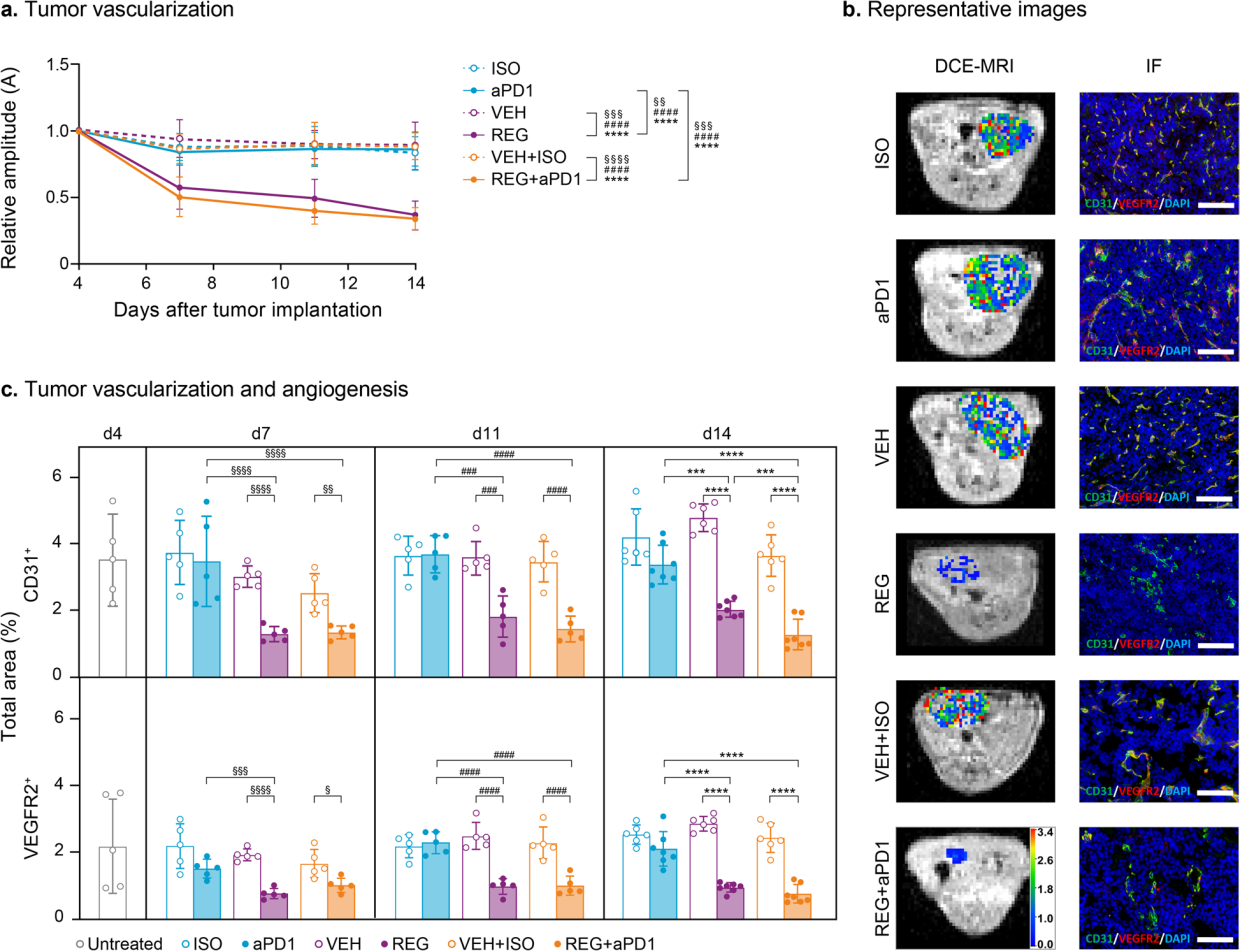
REG exerts potent anti-angiogenic effects in orthotopic CT26 tumors. **a** Tumor vascularization analyzed by DCE-MRI. Longitudinal effects on vascularization are shown by relative amplitude changes versus baseline (day 4). **b** Representative images. Left panel: T1-weighted MR images with overlayed amplitude parameter maps derived from DCE-MRI analyses (scale bar indicates arbitrary units); right panel: IF staining for CD31 (green), VEGFR2 (red) and nuclei (DAPI; blue). Yellow staining indicates overlapping signal for CD31 and VEGFR2. Scale bar: 100 µm. **c** Effects on tumor vasculature analyzed by immunofluorescence (IF) on frozen tumor sections for CD31 and VEGFR2 and quantified: mean values ± SD (*n* = 5–7) and individual values (dots). Statistical significance (^§^day 7; ^#^day 11; * day 14): *^/§/#^*p* < 0.05; **^/§§/##^*p* < 0.01; ***^/§§§/###^*p* < 0.001; ****^/§§§§/####^*p* < 0.0001

As REG has been previously shown to exert strong anti-angiogenic effects in various tumor models [8, 9, 33], we investigated the effects of REG in mono- and combination therapy on the vasculature of the orthotopic colon tumors. Tumor vascularization was determined longitudinally using DCE-MRI, and the Brix two-compartment model was used for pharmacokinetic analysis (Fig. 3a). The amplitude, an indicator of the relative distribution volume (RDV) of the contrast agent that reflects tumor vascularization, declined slightly in control groups from day 4 to 14 after implantation. In REG and REG + aPD1-treated tumors, a significant drop in RDV versus the respective control group was noticeable after 3 days of treatment. The RDV further declined in REG and REG + aPD1 groups, and differences to controls became even more pronounced on days 11 and 14, respectively. aPD1 alone had no effect on tumor vascularization (Fig. 3a, b panel “DCE-MRI”).

CD31 and VEGFR2 staining further validated the DCE-MRI data and the previously observed strong inhibitory effects of REG on tumor vascularization and angiogenesis [9]. Microvessels (CD31^+^ percentage of total area) were consistently reduced by approximately 50% in tumors of REG and REG + aPD1-treated animals by day 7 post-implantation versus respective controls, which aligned with the RDV profile and persisted at this level until day 14 after implantation. In line with the DCE-MRI data, aPD1 treatment had no effect on tumor angiogenesis (Fig. 3c, b panel “IF”).

Analysis of vessel maturation by quantifying αSMA^+^ vessels revealed no significant difference among the treatment groups (Supplementary Figure S2).

### Macrophages and regulatory T cells are significantly reduced in CT26 tumors in response to treatment with REG and REG + aPD1

**Fig. 4:**
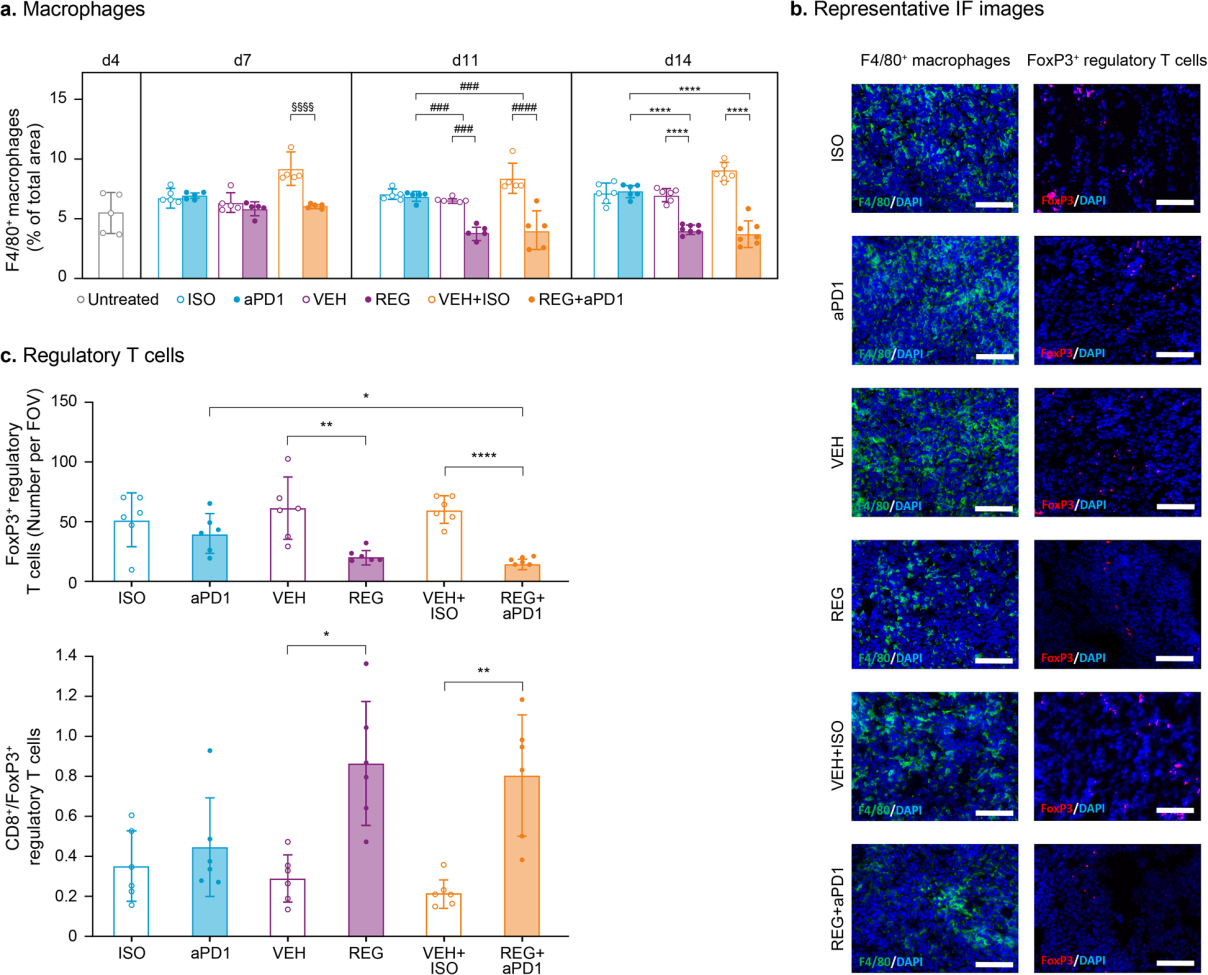
REG and REG + aPD1 significantly reduce intratumoral macrophages and Treg cells. **a** Staining of frozen tumor sections for macrophages (F4/80) and quantification: mean values ± SD (*n* = 5–7) and individual values (dots). **b** Representative immunofluorescence images. Left panel: staining for F4/80 in green; right panel: staining for FoxP3 in red. Nuclei are DAPI stained (blue). Scale bars: 100 µm. **c** Quantification of staining for Treg cells (FoxP3) on day 14 with mean values ± SD (*n* = 6–7) and individual values (dots) and ratio of CD8^+^ cytotoxic T cells to Treg cells (see Supplementary Figure S3a for quantification for CD8^+^). Statistical significance (^§^day 7; ^#^day 11; * day 14): *^/§/#^*p* < 0.05; **^/§§/##^*p* < 0.01; ***^/§§§/###^*p* < 0.001; ****^/§§§§/####^*p* < 0.0001

Our previous study demonstrated that REG significantly reduced intratumoral macrophages [9], and recent evidence indicates that PD1 blockade also influences tumor-associated macrophages (TAMs) [34]. Therefore, we investigated the effects of the various treatments on macrophages in the primary CT26 tumors. F4/80 staining and quantification revealed a significant and comparable reduction in macrophages in tumors treated with REG alone and REG + aPD1 (Fig. 4a, b panel “F4/80^+^ macrophages”). aPD1 alone had no effect on macrophage recruitment and infiltration into the primary colon tumors.

Next, we investigated intratumoral T cell populations, as ICIs such as aPD1 can have a direct effect on T cells. Quantification of intratumoral CD8^+^ and CD4^+^ T cell populations revealed no major changes among the treatment groups (Supplementary Figure S3). Interestingly, clear differences were observed when analyzing intratumoral FoxP3^+^ regulatory T (Treg) cells. While aPD1 only had a minimal effect on the number of intratumoral Treg cells, REG and REG + aPD1 induced significant and comparable reductions in Treg cell numbers on day 14 post-implantation compared with the control groups (Fig. 4c, b panel “FoxP3^+^ regulatory T cells”). These results revealed novel immune-modulatory effects of REG by inhibiting Treg cell infiltration. As CD8^+^ cell numbers were not affected by any of the treatments (Supplementary Figure S3a), significantly increased ratios of CD8^+^/FoxP3^+^ cells were observed in tumors in the REG and REG + aPD1 groups versus control-treated animals, whereas there was only a minor increase in the CD8^+^/FoxP3^+^ ratio in aPD1-treated animals (Fig. 4c). Thus, it is likely that the increased CD8^+^/FoxP3^+^ ratio, which is considered a measure of anti-tumor immunity, contributes to the anti-tumor effects of REG and REG + aPD1.

### Treatment with REG + aPD1 leads to prolonged CT26 tumor growth inhibition and liver metastasis control even after discontinuation of therapy

During continuous treatment for 10 days, the inhibitory effects of REG alone and REG + aPD1 on orthotopic colon tumor growth, vascularization, macrophage infiltration and metastasis were similar, indicating that the addition of aPD1 to REG did not increase the therapeutic benefit. However, long-term tumor-suppressing effects of ICIs have been observed in other indications such as melanoma [35]; therefore, we performed a post-therapeutic progression study to assess potential differences between the longer-term effects of REG and REG + aPD1.

**Fig. 5:**
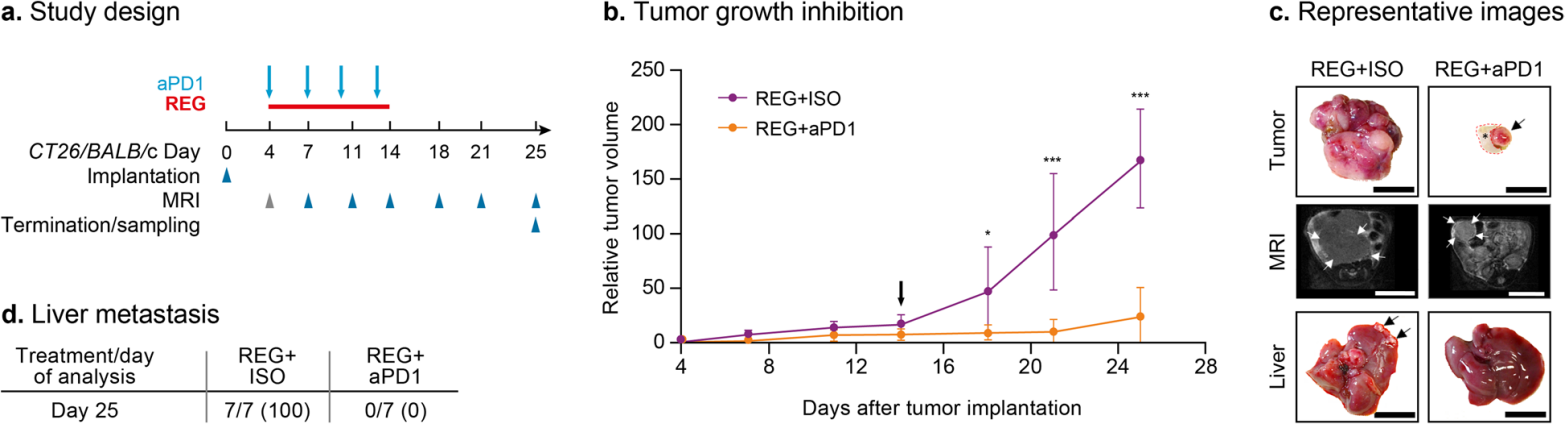
Addition of aPD1 to REG leads to sustained inhibition of tumor growth and metastasis after discontinuation of therapy. **a** Study design. Gray arrowhead indicates imaging before first treatment for baseline determination. **b** Tumor growth curves, mean RTV given as a percentage of the baseline volumes ± SD (*n* = 7). Black arrow indicates treatment stop. Statistical significance: *p < 0.05; ***p < 0.001. **c** Representative images. Top panel: dissected tumors (arrow indicates tumor; * indicates cecum tissue); middle panel: T2-weighted MR images, white arrows indicate tumors; bottom panel: livers with metastases indicated by black arrows. Scale bar: 10 mm (MRI and ex vivo). **d** Number of mice with liver metastases vs total number of mice and percentages (number in %)

Analogous to the efficacy study, animals received continuous treatment with REG + ISO or a combination of REG + aPD1 until day 14 after implantation. Subsequently, therapy was stopped, and tumor progression was monitored longitudinally by MRI until day 25 post-implantation (Fig. 5a).

Similar to the results of the efficacy study, tumor growth was efficiently inhibited by REG and REG + aPD1 during treatment until day 14 post-implantation. However, upon treatment discontinuation, tumors in mice that had previously received REG monotherapy regrew exponentially until the end of the experiment on day 25. By contrast, tumors in mice previously receiving REG + aPD1 remained largely unchanged until day 21, followed by a minor increase in the mean tumor volume between days 21 and 25 (Fig. 5b, c panels “tumor” and “MRI”; see Supplementary Figure S4a for tumor growth curves of individual mice).

In addition to the significant disparity in post-therapeutic tumor regrowth, ex vivo necroscopy on day 25 revealed major differences in the occurrence of liver metastases between the REG and REG + aPD1 treatment groups (Fig. 5d). Notably, while all mice had macroscopic liver metastases in the REG group, no mice in the REG + aPD1 group had observable liver metastases (Fig. 5c, panel “liver”, d).

**Fig. 6:**
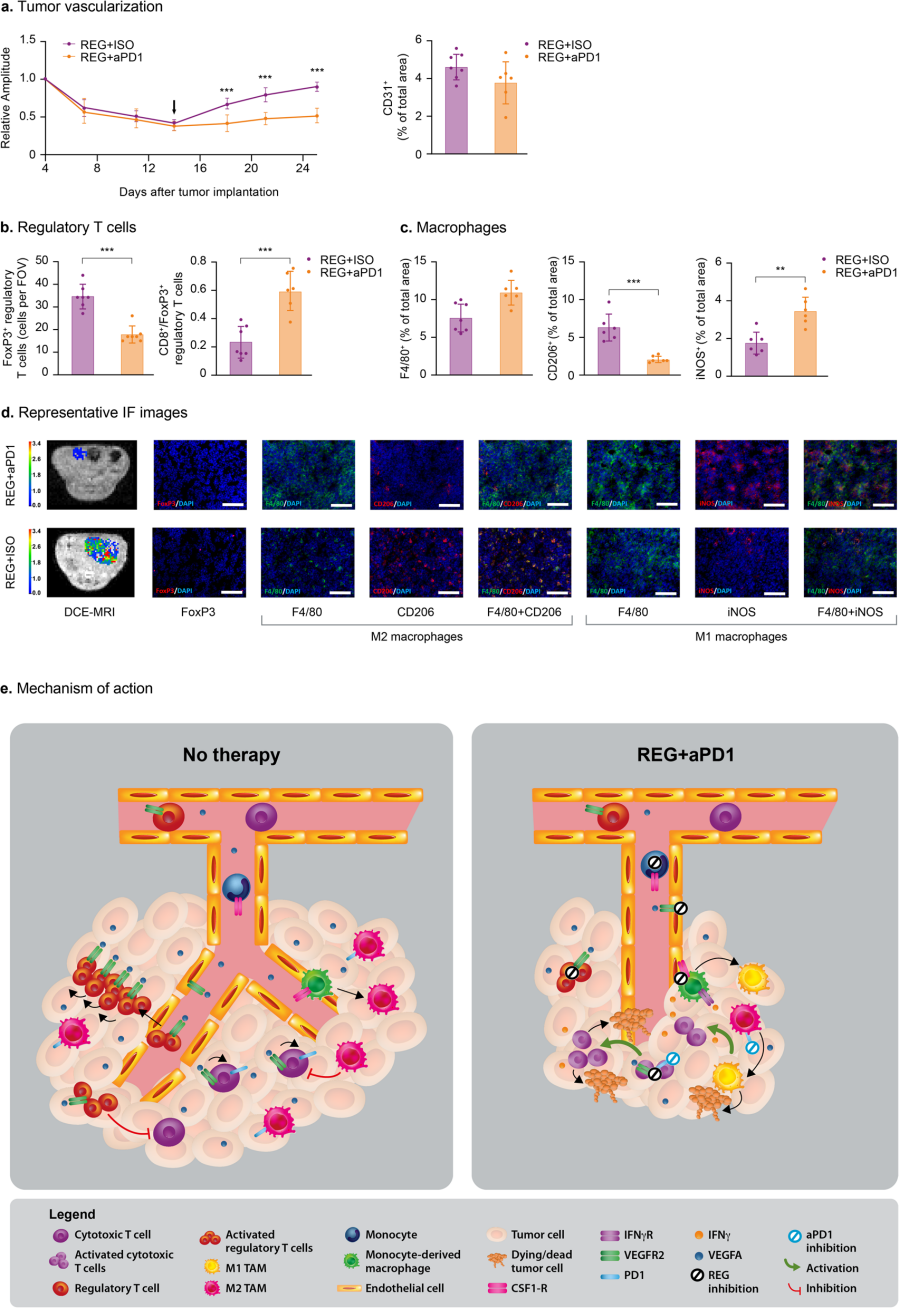
REG + aPD1 relieves the immunosuppressive tumor microenvironment and induces sustained suppression of tumor growth and metastasis. **a** Effects on tumor vasculature analyzed by DCE-MRI and CD31 staining. Longitudinal effects on vascularization are shown by relative amplitude changes versus baseline (day 4). Black arrow indicates treatment stop. **b** Effects on Treg cells. Quantification of immunostaining for FoxP3^+^ Treg cells and determination of the ratio of CD8^+^ cytotoxic T cells to Treg cells (see Supplementary Figure S4b for quantification of CD8^+^ cells). **c** Effects on macrophages and polarization. Quantification of total macrophages (F4/80), CD206 (M2) and iNOS (M1). Mean values ± SD (*n* = 6–7) and individual values (dots) are shown. *p < 0.05; **p < 0.01; ***p < 0.001. **d** Representative DCE-MRI and immunofluorescence images. Left panel: T1-weighted MR images with overlayed amplitude parameter maps from DCE-MRI analyses (scale bar indicates arbitrary units); panels 2–8: images of co-stainings as indicated by the antigen labels. Yellow staining indicates overlapping signals. Nuclei are DAPI stained (blue). Scale bars: 100 µm. **e** Mechanisms of sustained tumor growth inhibition by REG + aPD1. In untreated tumors, monocytes are recruited which differentiate to M2 macrophages. In addition, VEGFA promotes tumor angiogenesis, proliferation of Treg cells, and upregulation of PD1 on cytotoxic T cells. Together this creates an immunosuppressive tumor microenvironment that inhibits cytotoxic T cell activity, thus driving tumor growth and liver metastasis. Via inhibition of VEGFR and CSF1R signaling, REG reduces tumor vascularization and reduces the number of intratumoral macrophages and Treg cells. Blockade of PD1 by aPD1, which is likely enhanced by REG-mediated prevention of PD1 expression, leads to the activation of cytotoxic T cells, increases IFNγ expression, and induces tumor cell death. Importantly, REG and aPD1 synergize to induce sustained M1 polarization resulting in durable suppression of tumor growth and liver metastasis

Durable inhibition of tumor vascularization was only observed in the combination therapy group. The RDV decreased rapidly from the start of treatment, with reduction of ~ 60% on day 14 post-implantation in the REG and REG + aPD1 groups. However, after treatment cessation, the RDV returned to a level similar to the baseline value at treatment initiation in tumors of the REG group, while the RDV remained stably low in tumors of REG + aPD1-treated mice (Fig. 6a, d panel “DCE-MRI”). Differences between REG and REG + aPD1 groups were significant as early as 4 days after treatment discontinuation. Additionally, more microvessels were detected in REG tumors compared with REG + aPD1 tumors on day 25, albeit there was no statistically significant difference between the groups (Fig. 6a diagram, Supplementary Figure S4c). These results suggest that the addition of aPD1 to REG prevents rapid tumor regrowth by inducing changes in the tumor microenvironment.

To elucidate the mechanisms of sustained tumor growth inhibition, we analyzed immune cell infiltrates from tumors on day 25 post-implantation. The number of CD8^+^ and CD4^+^ T cells was only slightly increased in tumors exposed to REG + aPD1 combination versus REG alone (Supplementary Figure S4b); however, the number of intratumoral Treg cells was significantly lower in the REG + aPD1 group versus REG alone, which was associated with a significantly higher CD8^+^/FoxP3^+^ ratio in tumors in the combination therapy group (Fig. 6c, d panel “FoxP3”).

Importantly, analysis of macrophage recruitment in tumors on day 25 revealed a higher number of intratumoral macrophages in the REG + aPD1 group versus the REG group (Fig. 6c left diagram, d panels “F4/80”). Since macrophages can have either pro- or anti-tumorigenic effects depending on their differentiation state, we investigated the polarization of intratumoral macrophages. Staining for CD206 as a marker for pro-tumorigenic M2 macrophages revealed significantly decreased numbers of M2 polarized macrophages in tumors treated with REG + aPD1 versus REG monotherapy (Fig. 6c middle diagram, d panels “CD206” and “F4/80 + CD206”). Staining for iNOS, which is commonly expressed in anti-tumorigenic M1 macrophages, showed significantly more iNOS^+^ macrophages in REG + aPD1 versus REG + ISO tumors (Fig. 6c right diagram, d panels “iNOS” and “F4/80 + iNOS”). These findings provide evidence that the combination of REG + aPD1 leads to a sustained polarization of TAMs towards the M1 phenotype, which together with an increased CD8^+^/Treg cell ratio may contribute to the synergistic anti-tumor activity of REG + aPD1 compared with REG monotherapy.

## Discussion

Despite demonstrating some clinical efficacy, neither targeted anti-angiogenic agents nor immunotherapies show robust and durable clinical responses when used as single agents in CRC, the majority of which are pMMR/MSS tumors. To improve the therapeutic outcomes in patients with CRC, combinations of both anti-angiogenic agents and immunotherapies are currently being explored. We investigated REG and its combination with aPD1 preclinically to assess anti-tumor effects and gain mechanistic insights. We used two syngeneic CRC mouse models, MC38 and CT26, with different mutational and microsatellite stability profiles [24, 25]. As previously reported [25, 36], aPD1 alone was more effective in MC38 tumors, which have a higher mutational load than CT26 tumors [25, 37]. This is in line with the better clinical responses to ICI monotherapy observed in patients with MSI/dMMR CRCs compared with patients who have MSS/pMMR CRC [38].

REG + aPD1 significantly improved anti-tumor activity in the subcutaneous MC38 and orthotopic CT26 model. In CT26 tumors, inhibition of tumor growth and liver metastasis by REG alone (30 mg/kg) was already very potent; however, the therapeutic benefit of REG + aPD1 became evident after discontinuation of treatment. After treatment was stopped, the anti-tumorigenic effects of REG were rather rapidly abrogated, but the combination of REG + aPD1 significantly prolonged tumor growth inhibition and led to sustained suppression of liver metastasis.

As aPD1 alone inhibited tumor growth only moderately during continuous treatment in CT26 tumors, it is unlikely that aPD1, because of a higher metabolic stability than REG, is solely responsible for the prolonged tumor growth inhibition after treatment discontinuation. To investigate the mechanisms behind the efficient and sustained tumor suppression induced by REG + aPD1, angiogenesis- and immune-related analyses were performed.

As shown in previous studies, REG alone demonstrated anti-angiogenic effects and significantly inhibited the recruitment of TAMs [7, 9]. Additionally, REG reduced the number of intratumoral Treg cells, which is a novel finding of this study, and indeed this may contribute to the anti-immunosuppressive effects (Fig. 6e). Mechanistically, this can be explained by inhibition of VEGFA/VEGFR signaling, as VEGFA enhances Treg cell proliferation [39] and increases the expression of surface proteins on endothelial cells that promote Treg cell trafficking [40]. The REG-mediated reduction of TAMs, which secrete factors that attract Treg cells, may also contribute to this effect. Other anti-angiogenic agents, such as the multi-tyrosine kinase inhibitor sunitinib or the anti-VEGFA antibody bevacizumab, have been shown to induce similar Treg cell-reducing effects in subcutaneous CT26 tumors and in the peripheral blood of patients with metastatic CRC [39]. In line with our findings in CT26 colon tumors, patients with gastric cancer who responded to REG + aPD1 therapy showed reduced levels of intratumoral Treg cells [23]. In contrast, aPD1 alone had little to no effect on tumor angiogenesis, TAMs, and intratumoral Treg cell numbers; however, aPD1 increased the levels of intratumoral IFNγ, which is a pharmacodynamic marker for cytotoxic T cell activity, whereas IFNγ was not affected by REG. Thus, the relief of immunosuppression induced by REG together with the reactivation of cytotoxic T cells by aPD1 may provide an explanation for the synergistic effects of REG + aPD1 (Fig. 6e).

Treg cell reduction and blockade of tumor vascularization by REG alone did not persist when therapy was stopped, whereas REG + aPD1 induced durable effects, as demonstrated by the significant delay in tumor revascularization and Treg reinfiltration between day 14 and day 25 and the trend towards higher intratumoral levels of CD4^+^ and CD8^+^ T cells. By contrast, inhibition of macrophage recruitment was not sustained by REG or REG + aPD1, and the macrophages reached even higher levels in tumors treated with REG + aPD1 10 days after treatment discontinuation, although the levels were similar compared with REG monotherapy at the end of treatment on day 14. On day 25, the intratumoral macrophages were predominantly iNOS^+^ and considered M1 in the REG + aPD1 group, whereas the majority of macrophages were CD206^+^ and considered M2 in the REG group. This finding provides further insight into the mechanism of the durable tumor suppression observed with REG + aPD1 therapy. M2 macrophages secrete pro-tumorigenic, pro-angiogenic, and immunosuppressive factors such as IL10, TGFβ, and VEGFA; whereas M1 macrophages produce pro-inflammatory cytokines such as TNFα, IL1, IL12, and IFNγ, and reactive oxygen species, which prevent tumor growth [41]. Our findings suggest that REG and aPD1 act synergistically to induce sustained M1 macrophage polarization, as this phenomenon was only observed with the combination therapy (Fig. 6e). Mechanistically, one possibility is that REG directs monocyte differentiation towards M1 macrophages by inhibiting CSF1R, the receptor of M-CSF, which normally promotes differentiation into M2 macrophages. However, this effect of REG on macrophages was not long lasting and was diminished after REG treatment was discontinued, resulting in the predominance of M2 polarized macrophages 10 days after treatment discontinuation. Thus, sustained M1 macrophage polarization is also dependent on the action of aPD1. PD1 blockade leads to the release of IFNγ from reactivated T cells, which is a major inducer of M1 polarization [42]. This is supported by the significantly increased levels of IFNγ measured in MC38 tumors treated with aPD1 but not in those that received REG treatment. Nevertheless, REG, as a potent inhibitor of VEGFA-signaling, may further add to reactivation of T cells, not only by reducing Treg cells but also by preventing VEGFA-induced PD1 expression in cytotoxic T cells [43], although this contribution was not measurable in the context of total intratumoral IFNγ levels. In addition to promoting M1 polarization via T cell activation, PD1 blockade can also directly modulate macrophage activity and polarization. PD1 was found to be expressed on intratumoral M2 macrophages and to dampen their phagocytic activity towards tumor cells [34]. aPD1 was shown to reactivate the phagocytotic potency of TAMs, which slowed tumor growth and increased survival time in mice [34]. In addition, a recent study in an osteosarcoma model showed that aPD1 enhanced infiltration of macrophages into the tumor and induced a shift towards M1 polarization leading to reduced lung metastasis [44]. These results are in line with our observations in colon tumors and provide evidence that macrophage modulation may be an important mechanism of the anti-tumor activity of aPD1. Thus, REG and aPD1 most likely synergize to differentiate macrophages into a more stable, anti-tumorigenic M1 subtype, which persists for some time in the absence of treatment (Fig. 6e). Recent research shows that macrophage differentiation is highly complex, and an increasing number of subtypes are being identified [45], which requires further investigation for a more comprehensive understanding.

When considering drug dosing for an efficient combination therapy, REG administered at 3 mg/kg induced only moderate tumor growth inhibition in the subcutaneous MC38 model, but the anti-tumor activity was significantly enhanced in combination with aPD1. The 3 mg/kg REG dose corresponds to less than half of the clinically applied monotherapy dose of 160 mg per day, and therefore, this may indicate that REG can be administered in combination at a dose below 160 mg. This dose may still be sufficient to inhibit target kinases, contributing to the combination effect with aPD1 and perhaps with other ICIs. In fact, the combination of nivolumab with REG 120 mg or 80 mg demonstrated potent anti-tumor activity in CRC and gastric cancer in a phase I study [23]. Further investigation is warranted to identify an optimal clinical dose of REG in combination with ICIs and/or to identify agents which could further enhance the anti-tumor activity of such a combination therapy. Various immunomodulatory mechanisms are under consideration including for example arginine metabolism [46–48] and β-glucan, an innate immune activator, is even in advanced clinical testing for combination with cetuximab in selected CRC patients [49].

## Conclusions

Our findings demonstrate that REG + aPD1 exerts significant beneficial anti-tumor activity versus single agents, leading to sustained inhibition of colon cancer growth and liver metastasis. Sustained tumor suppression by REG + aPD1 can be explained by their synergistic immunomodulatory effects with the primary mechanism of M1 macrophage differentiation and activation and continuous inhibition of Treg cell infiltration, leading to the activation of cytotoxic T cells and efficient killing of tumor cells (Fig. 6e). Therefore, our findings strongly encourage clinical investigation of REG + aPD1 in CRC and other tumor types.

## Supplementary Information

**Additional file 1.** Supplementary Methods.

**Additional file 2:Figure S1.** Growth curves of individual orthotopic CT26 tumors (spider plot) in the efficacy study. **Figure S2.** Effects of treatments on tumor blood vessel normalization. **Figure S3.** Effects of treatments on intratumoral CD4^+^ and CD8^+^ T cells. **Figure S4.** Spider plot of individual tumor growth and vascular and cytotoxic T cell effects in post-therapeutic progression study.

## Data Availability

All methods and materials used are described in the manuscript and data can be obtained from the corresponding authors upon request.

## Abbreviations

aPD1: Anti programmed cell death protein 1 antibody
BMDM: Bone marrow-derived monocytes
CRC: Colorectal cancer
CSF1-R: Colony-stimulating factor 1 receptor
d: Day
DAPI: 4′,6-Diamidino-2-phenylindole
DMEM: Dulbecco's modified Eagle's medium
DCE-MRI: Dynamic contrast-enhanced MRI
dMMR: Deficient in mismatch repair
EC: Endothelial cell
EGFR: Epidermal growth factor receptor
FGFR: Fibroblast growth factor receptor
FITC: Fluorescein isothiocyanate
FLASH: Fast low angle shot
FOV: Field of view
FoxP3: Forkhead box P3
GAPDH: Glycerinaldehyde-3 phosphate-dehydrogenase
ICI: Immune checkpoint inhibitor
IF: Immunofluorescence
IL: interleukin
iNOS: Inducible nitric oxide synthase
i.p.: Intraperitoneal
ISO: Isotype control antibody
kg: Kilogram
MDM: Monocyte-derived macrophage
MRI: Magnetic resonance imaging
MSI: Microsatellite instable
MSS: Microsatellite stable
pMMR: Proficient in mismatch repair
NSA: Number of signal averages
PEG: Polyethylene glycol
PDGFR: Platelet-derived growth factor receptor
RDV: Relative distribution volume
REG: Regorafenib
rIgG2a: Rat immunoglobulin gamma 2A
RTV: Relative tumor volume
SMA: Smooth muscle actin
VEGFR1/2/3: Vascular endothelial cell growth factor receptor -1, -2, -3
VEH: Vehicle
TAM: Tumor-associated macrophage
TC: Tumor cell
TE: Echo time
TR: Repetition time
TUNEL: TdT-mediated dUTP-biotin nick end labelling
Treg: Regulatory T cell
