# Supplementary material for: Regorafenib enhances anti-PD1 immunotherapy efficacy in murine colorectal cancers and their combination prevents tumor regrowth
Source: J Exp Clin Cancer Res. 2021 Sep 13;40:288. doi: 10.1186/s13046-021-02043-0 (PMC8436536; doi:10.1186/s13046-021-02043-0)
Supplement: Supplementary file 1 — Additional file 1. Supplementary Methods. [file 13046_2021_2043_MOESM1_ESM.docx]

**Supplementary Methods**

*Detailed information on the MRI sequences*

T1-weighted Rapid Acquisition with Relaxation Enhancement (RARE) spin echo sequence:

Repetition time: TR = 579ms; echo time: TE = 12ms; RARE factor = 2; flip angle = 90°; number of signal averages: NSA= 8; field of view: FOV = 35mm x 35mm; matrix size = 256 x 256; slice thickness = 1.25mm; voxel size = 0.14mm x 0.14mm x 1.25mm).

T2-weighted Rapid Acquisition with Relaxation Enhancement (RARE) spin echo sequence:

Repetition time: TR = 1681ms; echo time: TE = 84ms; RARE factor = 8; flip angle = 90°; number of signal averages: NSA= 8; field of view: FOV = 35mm x 35mm; matrix size = 192 x 192; slice thickness = 1.25mm; voxel size = 0.18mm x 0.18mm x 1.25mm).

T1-weighted saturation recovery Fast Low Angle Shot (FLASH) sequence for DCE-MRI:

Repetition time: TR = 118ms; echo time: TE = 6ms; flip angle = 30°; number of signal averages: NSA= 1; Field of view: FOV = 30mm x 30mm; matrix size = 64 x 64; slice thickness = 1.25mm; voxel size = 0.50mm x 0.50mm x 1.25mm).

*Antibodies for immunofluorescent stainings and quantification of the stainings*

The following primary antibodies were used for immunofluorescent stainings: Antibodies against CD31 (rat anti-mouse, BD Biosciences, catalog #553370) and VEGFR2 (goat anti-mouse, R&D Systems, catalog #AF644) were used to assess tumor vessels and angiogenesis. For the analysis of vessel maturation an antibody against α-smooth muscle actin was used (biotinylated anti α-SMA, Progen, catalog #BK61501). Apoptosis was detected using the 'In Situ Cell Death Detection Kit, TMR red' (Roche Diagnostics GmbH, catalog #45-12156792910). Macrophages were stained with a FITC labeled rat anti-mouse F4/80-antibody (AbD Serotec, catalog #MCA497GA). CD4-positive T cells were stained with a rabbit anti-mouse CD4 antibody (GK 1.5, Invitrogen, catalog #MA1-146), CD8-positive T cells with a biotinylated rat anti-mouse CD8a antibody (53-6.7, Invitrogen, catalog **#**13-0081-82). For Treg cells, a rat anti-mouse FoxP3 antibody (eBioscience, catalog #14-5773-82) was used. M2-polarization was assessed by staining with a rat anti-mouse CD206 antibody (Acris, catalog #SM1857P). M1-polarization was detected using an antibody against inducible nitric oxide synthase (rabbit anti mouse iNOS, Abcam, catalog #ab15323). For visualization, donkey anti-goat-Cy3 antibody (Dianova, catalog #705-166-147), donkey anti-rabbit-Cy3 antibody (Dianova, catalog #711-166-152), donkey anti-rat Alexa Fluor 488 antibody (Dianova, catalog #712-546-153), donkey anti-rabbit Alexa Fluor 488 antibody (Dianova, catalog #711-546-152) and streptavidin-Cy3 (Promokine, catalog #PK-PF590-6-01) were used. Cell nuclei were counterstained by 4′,6-diamidino-2-phenylindole (DAPI, 0.5 mg/mL; Invitrogen, catalog #5748).

Microvessels were analyzed by determining the fraction of CD31 per area. Angiogenic activity was determined by calculating the ratio of the VEGFR2^+^ area to the CD31^+^ area. Apoptosis was evaluated by quantifying the terminal deoxynucleotidyl transferase–mediated dUTP nick end labeling (TUNEL)-positive area fraction. The percentage of SMA^+^ vessels was calculated by dividing the number of SMA^+^ vessels by the number of total CD31^+^ vessels; both were counted manually. The number of CD8^+^ T cells and the number of FoxP3^+^ T cells were determined by manual counting of positive cells per field of view (FOV), respectively. CD4^+^ T cells and macrophages were quantified by determining the CD4 and F4/80 positive area fractions, respectively. M1 and M2 macrophage polarization was determined by calculation of the iNOS and CD206 positive area fractions, respectively.

**Legends to Supplementary Figures**

**Fig. S1**. Growth curves of individual orthotopic CT26 tumors (spider blot) in the efficacy study. RTV determined by MRI measurements are presented as a percentage of the baseline volumes. Top: aPD1; middle: REG; bottom: REG+aPD1.

**Fig. S2.** Effects of treatments on tumor blood vessel normalization. **A,** Vessel normalization. Quantification of CD31 and αSMA (pericyte marker) immunostaining of tumor cryosections on day 14 with mean values ± SD (n=5–7) and individual values (dots). **B,** Representative immunofluorescence images. Staining for CD31 in green, αSMA in red, and nuclei in blue (DAPI). Yellow staining indicates overlapping signals for CD31 and αSMA. Scale bar: 100 µm.

F**ig. S3.** Effects of treatments on intratumoral CD4^+^ and CD8^+^ T cells. **A,** Quantification of T cells from cryosections of tumors on day 14 stained for CD4 and CD8. Mean values ± SD (n=6–7) and individual values (dots) are shown. **B,** Representative images from immunostaining for CD4 and CD8 in red and for nuclei in blue (DAPI). Scale bar: 100 µm. FOV, field of view.

**Fig. S4.** Spider blot of individual tumor growth and vascular and cytotoxic T cell effects in post-therapeutic progression study. **A,** Growth curves of individual tumors; RTV given as a percentage of the baseline volumes. **B,** Effects on intratumoral CD4^+^ and CD8^+^ T cells. Quantification of immunostaining for CD4 and CD8 from tumor sections on day 25. Mean values ± SD (n=6–7) and individual values (dots) are shown. **C,** Representative immunofluorescence images. Top panel: CD31 staining in green; middle panel: CD8 staining in red; bottom panel: CD4 staining in red. Nuclei are DAPI stained (blue). Scale bar: 100 µm. FOV, field of view
