# Supplementary material for: Regorafenib enhances anti-PD1 immunotherapy efficacy in murine colorectal cancers and their combination prevents tumor regrowth
Source: J Exp Clin Cancer Res. 2021 Sep 13;40:288. doi: 10.1186/s13046-021-02043-0 (PMC8436536; doi:10.1186/s13046-021-02043-0)

Additional File 2: Figure S1

Tumor growth

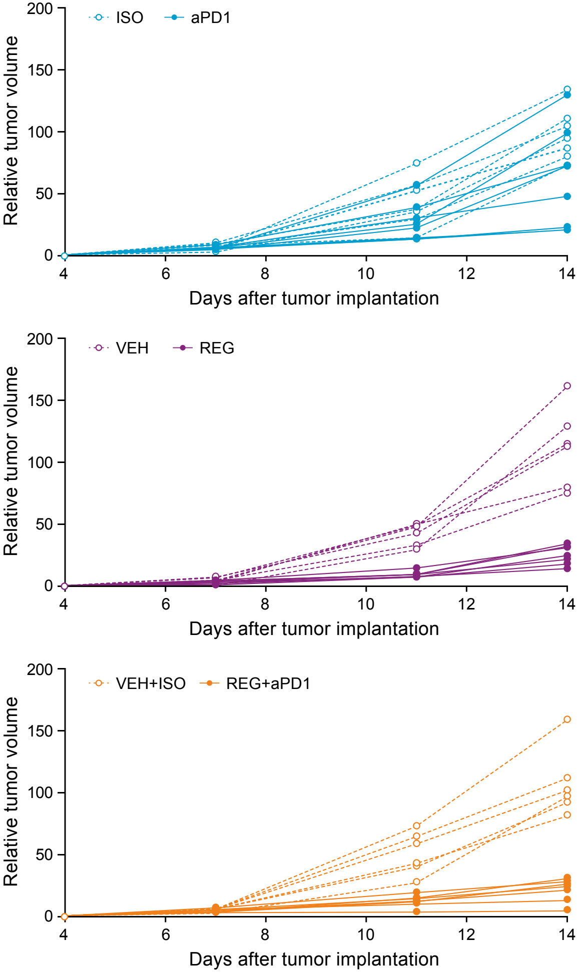

Additional File 3: Figure S2

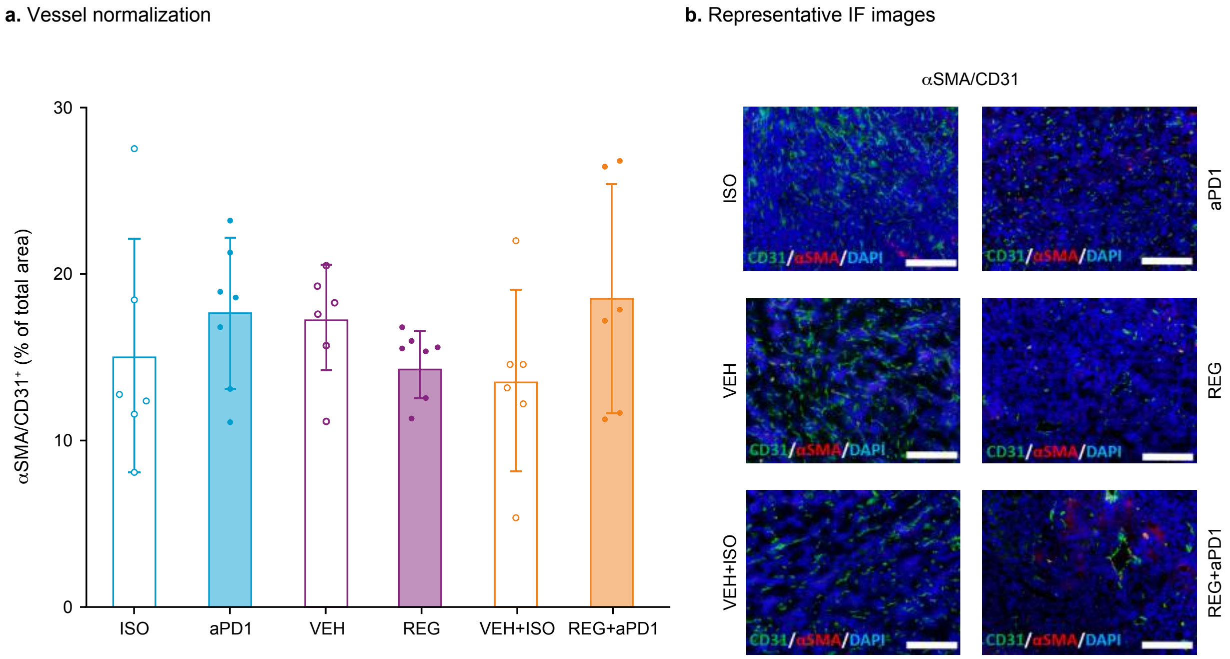

Additional File 4: Figure S3

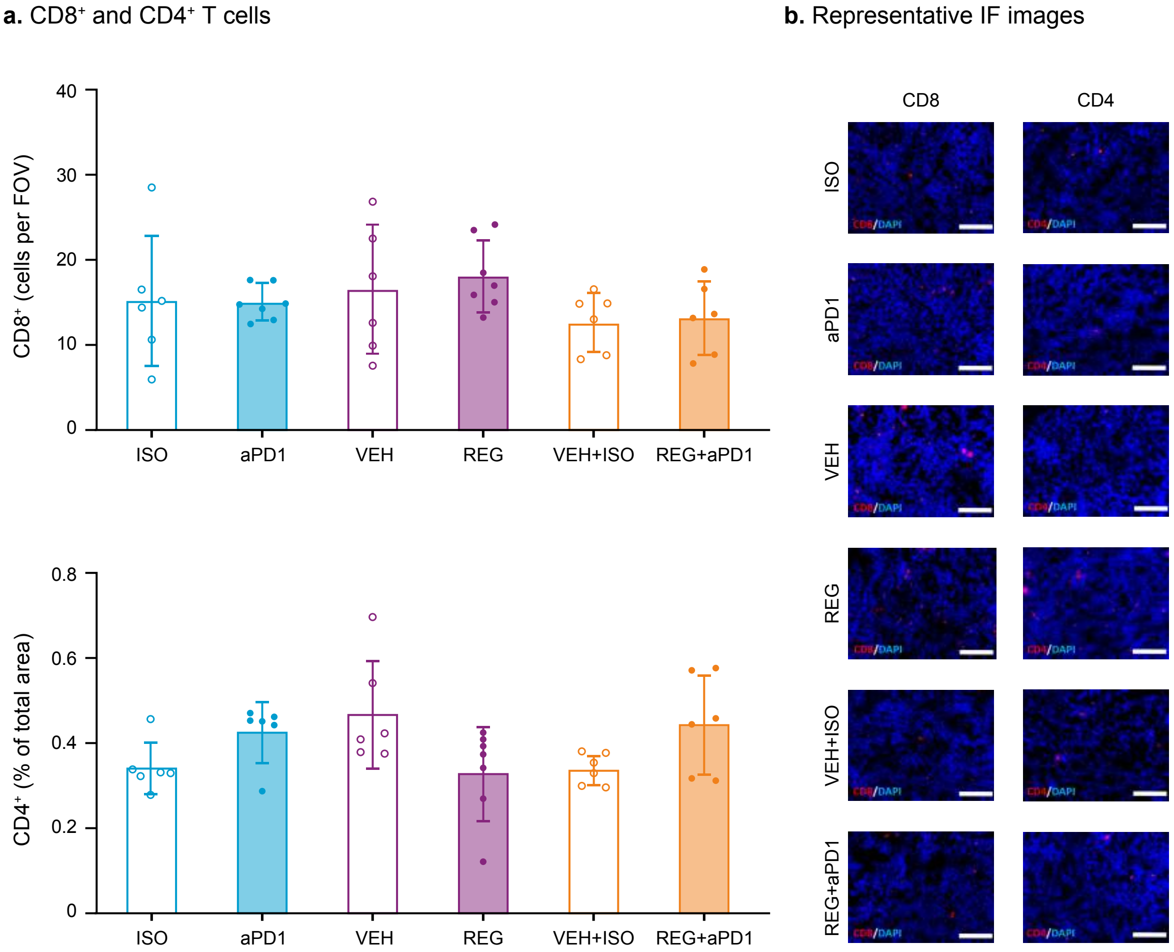

Additional File 5: Figure S4

a. Tumor growth

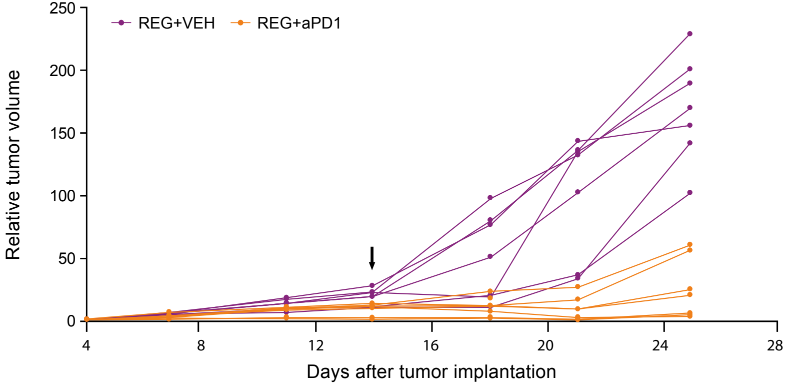

c. Representative IF images

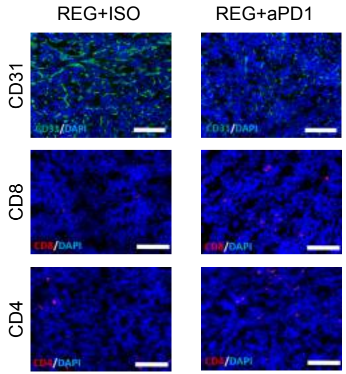

b. CD8⁺ and CD4⁺ T cells

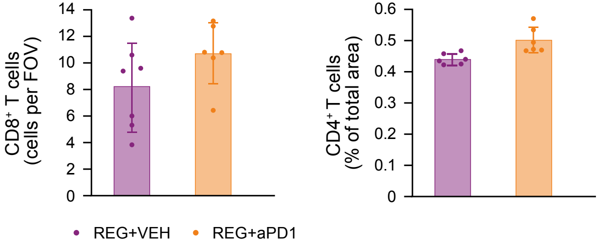

Supplement: Supplementary file 2 — Additional file 2:Figure S1. Growth curves of individual orthotopic CT26 tumors (spider plot) in the efficacy study. Figure S2. Effects of treatments on tumor blood vessel normalization. Figure S3. Effects of treatments on intratumoral CD4+ and CD8+ T cells. Figure S4. Spider plot of individual tumor growth and vascular and cytotoxic T cell effects in post-therapeutic progression study. [file 13046_2021_2043_MOESM2_ESM.pdf]
